# A new microdispersed albumin derivative potentially useful for radio-guided surgery of occult breast cancer lesions

**DOI:** 10.1038/s41598-019-42014-2

**Published:** 2019-04-04

**Authors:** Gabriele Caviglioli, Marco Chinol, Sara Baldassari, Lucia Garaboldi, Guendalina Zuccari, Andrea Petretto, Giuliana Drava, Chiara Sinico, Giovanni Paganelli

**Affiliations:** 1 Department of Pharmacy (DIFAR), University of Genova, 16148 Genova, Italy; 2 Division of Nuclear Medicine, European Institute of Oncology, 20141 Milano, Italy; 3 Core Facilities-Proteomics Laboratory, Istituto Giannina Gaslini, 16147 Genova, Italy; 4 Department of Life and Environmental Sciences, University of Cagliari, 09124 Cagliari, Italy

## Abstract

This paper describes a new nuclear imaging agent, 2-(4-isothiocyanatobenzyl)−1,4,7,10-tetraazacyclododecane-1,4,7,10-tetraacetic acid of human albumin (HAC), potentially suitable for application in the Radio-guided Occult Lesion Localization (ROLL) of non-palpable mammalian cancerous lesions, as a tool to overtake the short radio-signal half-life of the technetium-99m based radiopharmaceutical currently used. This conjugate is a microsized powder aggregate, water-insoluble between pH 3 and 8.5, obtained by conjugating the protein with the macrocyclic chelating agent DOTA through a one-pot reaction in aqueous medium. The product has been fully characterized and is stable to the thermal conditions adopted for labeling; after radiolabeling with longer half-life radionuclides such as ^177^Lu or ^111^In, it has shown radiochemical purity (RCP) >90% and resulted stable when stored in saline or plasma for 6 days at 37 °C. A μPET/CT study, performed *in vivo* on adult female rats, showed that the radioactivity of HAC labeled with ^64^Cu remained located in the mammary glands for at least 40 h, without diffusion or drainage in healthy tissues or in the lymphatic circulation. This new imaging agent might make the ROLL procedure more accessible, safe and flexible, promoting a significant time and cost reduction of this intervention. Moreover, HAC might also be used in other radio-guided surgical procedures in oncology.

## Introduction

Breast cancer is the second most common cancer in the world and ranks as the second cause of death from cancer overall after lung cancer; moreover, it is the leading cause of cancer death among women worldwide^1^. The estimated incidence in the 28 EU countries in 2018 was 144.9 new occurrences, with an estimated number of deaths of 31.4 for 100,000 inhabitants (age-standardized rate, from ECIS - European Cancer Information System). The lifetime risk of developing breast cancer for European women is 1 in 8.

As conclusively demonstrated, the early eradication of small, still non-palpable breast malignancies, which account for approximately one-third of all diagnosed breast cancers^2^, decreases the mortality; hence, the current goal is the surgical removal of occult lesions as soon as they are detected, to avoid relapses or metastases.

Excluding the clinical conditions requiring mastectomy, in the other cases only the non-palpable lesions considered possible pre-invasive tumors must be removed. The surgical excision, while eliminating all traces of the cancer lesion, should possibly be conservative, to maintain the integrity of the woman’s body and the gland functionality. For this reason, a localization technique is necessary for the surgeon to find the suspect lesion and remove the smallest piece of healthy tissue containing it.

Different techniques have been developed with this purpose, including carbon marking and wire-guided localization, but the efficiency, safety and furthermore the economic benefits of ROLL (Radio-guided Occult Lesion Localization) have been confirmed by several meta-analyses^3–7^.

Lovrics *et al*.^3^, performing a systematic review of radio-guided versus wire-guided surgery, concluded that the radio-guided techniques yielded lower positive margin and re-operation rates, moreover improving cosmetic outcomes. Besides, wire-guided surgery can cause the displacement or deviation of the wire and patient discomfort.

ROLL, as currently used, was developed at the European Institute of Oncology (IEO) in Milan in 1996^8^. It involves the inoculation of a gamma-emitting radiolabeled dispersion of human albumin macroaggregates (MAA) into the non-palpable lesion, by ultrasound guide or by a mammographic stereotaxic system. Within 24 h, by means of an intra-operatory gamma-probe, the lesion is surgically removed in a targeted way. The validity of ROLL is demonstrated by a mean excision success higher than 90%^9^. It has been also demonstrated that ROLL is safe from the radioprotection point of view, as it involves negligible risk for patients and hospital staff and does not require that special radioprotection measure be adopted by surgeon or pathologist^10^.

Recently the use of indocyanine green (ICG), a soluble fluorescent dye, has been considered a reliable and safe alternative to the gold standard method using colloidal ^99m^Tc-labeled albumin for the identification of sentinel nodes, in hospitals without nuclear medicinal facility^11^.

However, ICG cannot be a valid substitute of insoluble Tc-labeled MAA in the ROLL procedure, because this fluorescent dye is soluble and can migrate, drained by the lymphatic flow, out of the non-palpable lesion, making the removal of the tumor tissues less accurate.

The radiolabeled drug currently used in ROLL is a non-specific complex between insoluble macroaggregates of human albumin and ^99m^Tc, which is prepared at the time of use through marketed kits differing for aggregated human albumin, SnCl_2_ (included in the MAA powder), and preservative content. Briefly, the radiolabeling procedure of the kit is based on a red-ox reaction: when the MAA powder is mixed with a solution of sodium pertecnetate (Na^99m^TcO_4_), SnCl_2_, a strong reducing agent, converts ^99m^Tc(VII) into the ^99m^Tc(III) ion, which precipitates as ^99m^TcCl_3_ inside the MAA.

The MAA, described for the first time in 1966^12^, can be obtained through different patented procedures^13^, often differing only for some details. All these procedures require a common preparatory scheme that includes: denaturation of human albumin (HA) by exposure to extreme pH values; addition of a suitable amount of SnCl_2_; adjustment of pH close to the isoelectric point of denatured HA (approx. 5.3); subsequent alternating cycles of heating (up to 110 °C) and quick cooling that induce the precipitation of the MAA and their stabilization. These last steps influence the dimensional distribution of the aggregates^14^.

Unfortunately, the main limitation of ROLL is the short half-life (6.02 h) of ^99m^Tc, which implies that the surgical procedure must be carried out on the day of localization or, in any case, within 24 h. This determines a procedural timing conflict among the medical departments involved and prevents an efficient planning of the surgical interventions; socio-economic aspects must also be considered, because the usability of this procedure is limited only to those centers having the department of nuclear medicine and the breast cancer surgery unit. On the other hand, a radiopharmaceutical labeled with a gamma-emitting radionuclide with longer half-life, such as ^177^Lu or ^111^In, would allow to schedule the surgical procedures on several days and even to carry out the localization, as well as its verifications, as an outpatient exam in the days preceding surgery, reducing hospitalization times and costs. Although the direct labeling of MAA with a longer half-lived radionuclide, as ^111^In, has been obtained by Watanabe *et al*.^15^, the resulting complex was characterized by very poor stability, just 180 min. Therefore, our idea was to develop a technique to stably bind a long-lived radioisotope to an insoluble HA support. This paper concerns the development and characterization of a new microdispersed imaging agent suitable for the ROLL procedure, able to improve efficacy, safety and health-care system results.

## Results and Discussion

### HAC preparation

At the beginning of this study, we conceived to conjugate directly the MAA with a chelating agent able to bind radioisotopes with a longer half-life compared to technetium. Among the many derivatives, that form stable and kinetically inert complexes with di- and trivalent cations^16^, we selected 2-(4-isothiocyanatobenzyl)-1,4,7,10-tetraazacyclododecane-1,4,7,10-tetraacetic acid (*p*-SCN-Bn-DOTA), for its easy reaction with nucleophilic sites, which are present on the amino acid lateral chains of HA, under mild conditions in aqueous media^17^. Then, the direct reaction of MAA with *p*-SCN-Bn-DOTA was attempted, but with poor results in terms of yield and reproducibility (data not shown). In fact, the formation of the MAA is based on the cross-linking between amines and carboxylic groups present on the lateral chains of the protein, therefore free amino groups are likely to be minimally present on the surface of the MAA.

The same problem was encountered using HA nanospheres or microspheres, which could be considered interesting carriers for radiolabeled drugs. In fact, the architecture of these systems involves the interaction between carboxylic and amino groups of the protein, or the consumption of these functional sites due to the cross-linking reaction with a reticulating agent, which reduce the conjugation possibility. Moreover, microsphere formation involves complex multi-step procedures, and the use of oil phases, organic solvents, and reticulating agents, such as glutaraldehyde^14^, making these systems less pure and safe for therapeutic use, as well as expensive. The scant number of conjugated chelating moieties on the surface of HA microparticles caused a weak radioactive signal and, moreover, in the case of conjugation on HA nanoparticles, the system became drainable and then unsuitable for radiolocalization purposes^18,19^.

Thus, to increase the conjugation yield of *p*-SCN-Bn-DOTA on HA, we attempted to perform the conjugation by dissolving *p*-SCN-Bn-DOTA directly in a HA aqueous solution, planning a subsequent denaturation/precipitation step by applying the above-mentioned procedures. For this reaction, a commercial HA injectable solution (200 mg/mL), representing an easily accessible source of high quality HA, was employed, but also this procedure, even if applying different pH and temperatures, did not lead to significant conjugation yields. The reason was that HA, in authorized medicinal products, is obtained from human plasma through a consolidated extractive procedure^20^, using thermo-stabilizers such as sodium caprylate and sodium acetyl tryptophanate. In fact, the interaction between the carboxylic group of thermo-stabilizers and the amine function of HA lysines^21^ prevented the reaction of the latter with the isothiocyanate group of *p*-SCN-Bn-DOTA.

Hence, before using HA in the conjugation reaction, a purification procedure, by repeated washings with a high ionic strength solution followed by ultrafiltration, was carried out on commercial HA to free its reactive sites, yielding purified albumin (HAP) almost quantitatively.

Then, the subsequent conjugation reaction performed on HAP, in carbonate buffer at 40 °C, gave, after a few minutes, a plentiful white gelatinous precipitate. In order to exclude that this was due to albumin denaturation and aggregation, a sample of HAP was treated, under the same reaction conditions, with DOTA, i.e. the chelating portion of *p*-SCN-Bn-DOTA lacking the phenylisothiocyanate moiety: after a 20-h treatment at 40 °C, no solid phase formed, thus indicating that the insoluble product derived from HA interaction with the isothiocyanate portion of *p*-SCN-Bn-DOTA. Moreover, through this one-pot reaction in aqueous phase, not only the conjugation between DOTA and HA was obtained, but also the precipitation of insoluble microaggregates occurred, avoiding the denaturing process and its potential repercussions on the purity, safety and labeling efficiency of the final product.

### HAC characterization

This conjugate, named HAC, features insolubility in aqueous environment between pH 3.0 and pH 8.5. Noteworthy is the thermal behaviour of HAC: while purified albumin, if heated at 90 °C for 30 min (the typical radiolabeling conditions), coagulates, HAC remains dispersed in suspension. The different thermal behaviour has been also observed in HAP and HAC samples submitted to DSC up to 250 °C: although no significant differences were recorded in the DSC profiles and the colour of both changed due to baking, HAP formed a matrix, while HAC remained in powder state (Fig. S1a).

Besides, when both materials are freeze-dried, HAC remains in powder state, while HAP acquires a markedly different aspect, in the form of thin flakes (Fig. S1b).

Furthermore, the comparison of the IR spectra of HAC (Fig. S2a) and HAP (Fig. S2b) shows some differences, attributable to the introduction of the C=S group, indicating the formation of the thioureic bond during the conjugation reaction, which are the band enlargement around 1530 cm^−1^ due to the presence of C=S, less polar than C=O, the modifications in the 1350–1200 cm^−1^ spectral region, and, above all, the absorbance at 700 cm^−1^, specific of the C=S bond^22^. These spectral differences are reproducible and significantly relevant, although not particularly emphasized in terms of signal transmittance, as a result of the low degree of substitution of the small molecule of *p*-SCN-Bn-DOTA (MW 551.6 Da) in the HA macromolecule (MW 66,500 Da).

**Figure 1:**
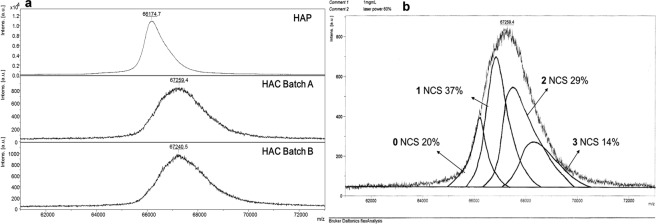
(**a**) MALDI-TOF analysis of lyophilized HAP and of two HAC microbatches; (**b**) deconvolution of HAC Batch A MALDI-TOF spectrum.

Notable results have been obtained by a MALDI-TOF mass spectrometry study that has allowed to establish that HAC is a mixture of albumin molecules containing a different number of conjugated DOTA residues. In fact, MALDI-TOF spectra of HAC showed an augmented mass (approx. 1070 Da) of the peak centroid with respect to HAP molecular weight (66,500 Da) (Fig. 1a). The shift and the widening of the peaks with m/z equal to 67,259 (Batch A) and m/z equal to 67,240 (Batch B), as shown in Fig. 1a, indicate a distribution of albumin molecules conjugated with a different number of DOTA, while the application of a geometric deconvolution method allowed to identify both the number of DOTA per albumin molecule and the relative abundance of the different conjugates (Fig. 1b). The mixture was composed of unconjugated albumin (15–20%), and mono- (35–37%), di- (29–30%) and three-substituted (14–20%) derivatives. However, considering the width of the peaks and the molecular weights of the possible conjugates, tetra, penta and hexa-substituted derivatives cannot be ruled out (Fig. S3).

To establish the site of conjugation, we performed comparative digestions of HAC and HAP using trypsin cleavage, and the amino acid sequences of the peptides thus obtained were identified by nanoLC-MS/MS. A quantitative proteomics software, designed for analyzing large mass-spectrometric data sets, was applied to screen the MS survey scans for target peptide masses in the hydrolyzed mixture from HAP and their modifications in the hydrolyzed mixtures from HAC samples.

**Figure 2:**
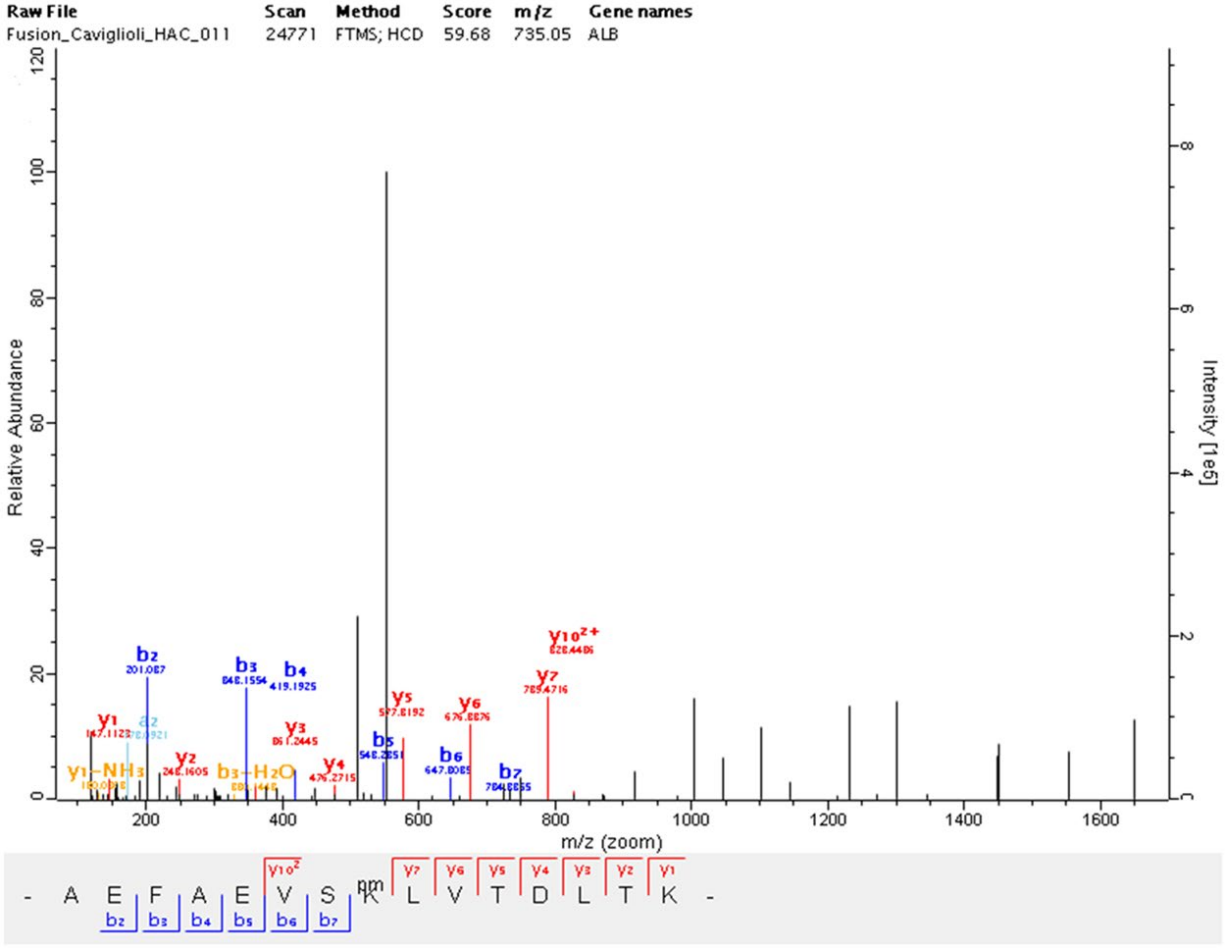
Annotated fragment spectrum of the AEFAEVSKLVTDLTK peptide identified for the presence of the *p*-SCN-Bn-DOTA residue. The corresponding sequence is displayed under the MS/MS spectrum and shows the position of the adduct (pm) on the central lysine site.

The mass increase of six detected amino acid sequences proved the binding of *p*-SCN-Bn-DOTA in correspondence to lysine residues to form thioureic derivatives (Table 1). Moreover, all the HAC-deriving peptides identified for the presence of a *p*-SCN-Bn-DOTA residue (552.213 Da) showed a longer chromatographic retention time with respect to the corresponding original peptides obtained by tryptic digestion of HAP. The MS/MS spectra (Figs. 2 and S4a–e) of each peptide show the lysine site of thioureic conjugation.

**Table 1:** Molecular weight and nanoLC-MS/MS retention times (Rt) of peptides deriving from tryptic digestion of HAP and HAC samples.

Peptide sequence*	Peptide mass in HAC digested mixture (Da)	Peptide mass in HAP digested mixture (Da)	Rt of HAC-deriving peptide (min)	Rt of HAP-deriving peptide (min)
AEFAEVSKLVTDLTK	2202.1000	1649.888	87.79	70.89
AFKAWAVAR	1570.7840	1018.571	64.30	50.07
KLVAASQAALGL	1692.8995	1140.687	74.67	62.82
KQTALVELVK	1679.9042	1127.691	65.67	54.07
KYLYEIAR	1606.7939	1054.581	63.61	50.56
NYAEAKDVFLGMFLYEYAR	2851.3111	2299.098	93.94	79.33

*Underlined letters indicate the conjugation site

Lastly, the average degree of substitution (DS) of HAC was determined through a modified and validated spectrophotometric UV assay^23,24^, measuring the decrease of absorbance at 656 nm of the blue-colored complex between arsenazo III and Pb(II), due to the concomitant formation of the highly stable Pb(II)-DOTA complex (Fig. S5).

In Table 2 the spectrophotometric measurement of the DOTA macrocycle content is reported as µmoles DOTA/mg HAC or as DS.

**Table 2:** DOTA macrocycle content, expressed as µmoles DOTA/mg HAC ± CI_95%_ or as average degree of substitution of DOTA on HA (DS = nDOTA/nHA), for four microbatches.

Batch #	DS nDOTA/nHA (mean ± CI_95%_)	µmoles DOTA/mg HAC (mean ± CI_95%_)
A	3.01 ± 0.64	0.044 ± 0.009
B	2.79 ± 0.53	0.041 ± 0.008
C	3.06 ± 0.66	0.045 ± 0.009
D	3.34 ± 0.68	0.049 ± 0.010

CI_95%_ = confidence interval at level of significance of 0.05%.

It is important to stress that the average DS value, calculated by the aforementioned spectrophotometric method, matches well with the MALDI-TOF and nanoLC-MS/MS evidences.

Reaction temperature and time influence the progress of the conjugation of HAC. The control of the reaction temperature is essential, as below 35 °C the reaction does not proceed significantly. The optimal reaction temperature ranges from 35 to 45 °C, and a higher temperature does not increase the conjugation yield. At 40 °C, the maximum HAC yield (60% w/w) was obtained with the reaction time lasting 16 h.

**Figure 3:**
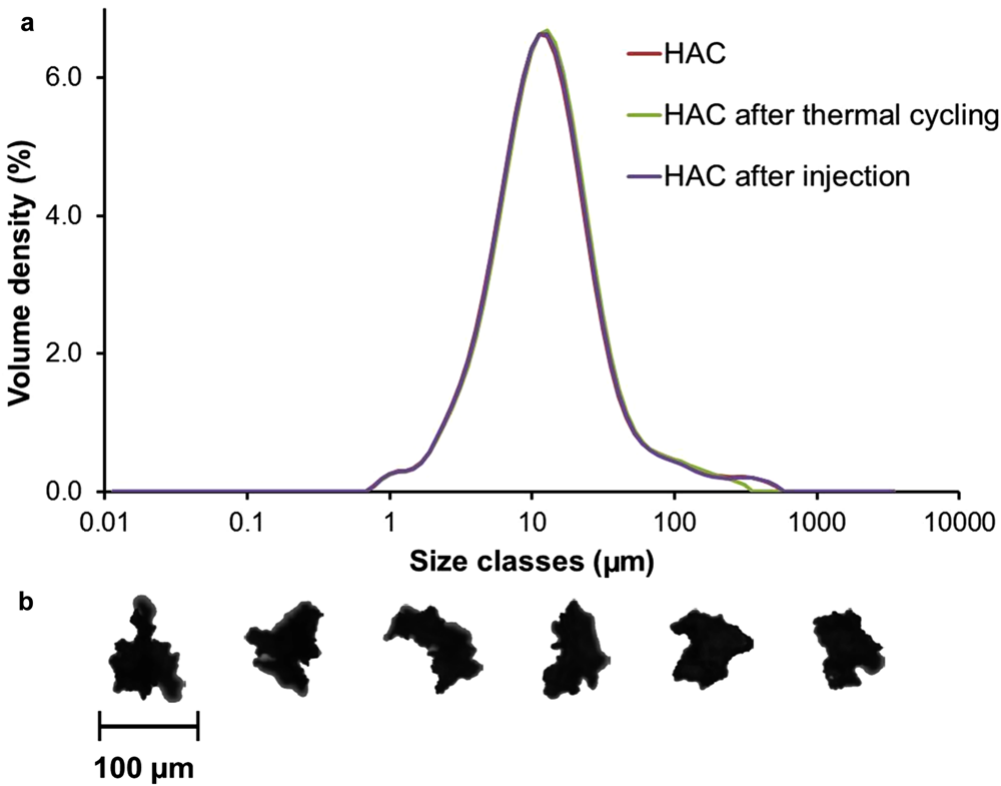
(**a**) Volume-weighed particle size distribution of HAC (Batch A) determined by laser diffraction as such, after thermal treatment and after injection; (**b**) shape of some HAC granules (Batch A) with approx. 100 µm diameter selected by an automated imaging system.

HAC particle size distribution has been studied by laser diffraction and 80% of the total volume of HAC aggregates resulted in the range from 4 to 44 µm (Table 3). The size distribution of the aggregates did not change when HAC suspensions were subjected to cycles of heating to 90 °C and rapid cooling to 5 °C, or when HAC suspension was passed through a 21-gauge needle (Fig. 3).

**Table 3:** Main size distribution parameters calculated from five HAC batches obtained by conjugation reaction performed at 40 °C for 16 h.

% volume under size	Size ± SD (µm)
10%	4.3 ± 0.0
50%	12.7 ± 0.1
90%	44.4 ± 3.2
99%	211 ± 14.0

An analysis by automated microscope imaging system showed high circularity (mean HS circularity 0.79) and low elongation (0.29) of the HAC microaggregates, suggesting an aggregate shape generally closer to sphericity than to acicular morphology; this seems to be confirmed by the planar image of a HAC sample in Figure 3b.

Importantly, HAC has been obtained in sterile form under aseptic conditions, starting from commercial HA formulations, as confirmed by a sterility control performed on different batches.

### HAC radiolabeling study

For labeling study, HAC samples were tested for complexation with non-radioactive lutetium (^175^Lu) and with radioisotopes (^177^Lu, ^111^In). The complexation procedure with non-radioactive Lu allowed to calculate the incorporation yield of the metal in HAC, carrying out the process at the same conditions used for radioisotopic labeling. The mean yield of the purified and freeze-dried complex HAC-Lu(III), calculated on the basis of the weight of HAC used, was 100.0 ± 0.3% (w/w) (CI_95_, n = 5). By using Lu/HAC molar ratios between 130/1 and 100/1 for complexation, the incorporation efficiency of Lu, determined by ICP-OES on the basis of the available moles of DOTA per mg of HAC, ranged between 40% and 60% of the weight of lutetium used. Such yield corresponded to an incorporation range between 20 and 26 nmoles of Lu/mg HAC.

These measurements, besides being mandatory to assess HAC metal complexation efficiency under the labeling conditions, confirmed that HAC, although not soluble in aqueous phase, was able to exert its complexing action even in suspension, i.e. in a heterogeneous phase. By the same test, HAC has demonstrated to preserve its complexing action after freeze-drying and reconstitution as aqueous suspension.

Next, HAC radiolabeling with ^177^Lu or ^111^In has been performed both in suspension (acetate buffer pH 5.0) and after reconstitution of the lyophilizate, obtaining a product with specific activity of 1 mCi/mg and radiochemical purity always higher than 90%.

Table S1 and Figure S6 show the radiochemical purity of HAC microbatches labeled with ^111^In and ^177^Lu.

The *in vitro* stability of HAC labeled with ^177^Lu and ^111^In was assessed in saline suspension and the product resulted stable to radiolysis (RCP > 95%) for at least 6 days after labeling.

HAC labeled with ^177^Lu and ^111^In has also shown to be stable in human plasma suspension stored at 37 °C for 6 days (RCP > 95%)^25^.

### µPET and µCT *in vivo* study

Regarding the *in vivo* capability of HAC to be retained in the mammary glands, a viable µPET (positron emission tomography) and a µCT (X-ray computed tomography) imaging study have been performed on two adult female rats. In this case, HAC was labeled with ^64^Cu (t_1/2_ = 12.7 h), a positron-emitting radioisotope producible in large quantities and with high specific activity in biomedical cyclotrons, and thus widely used for preclinical *in vivo* studies^26,27^.

**Figure 4:**
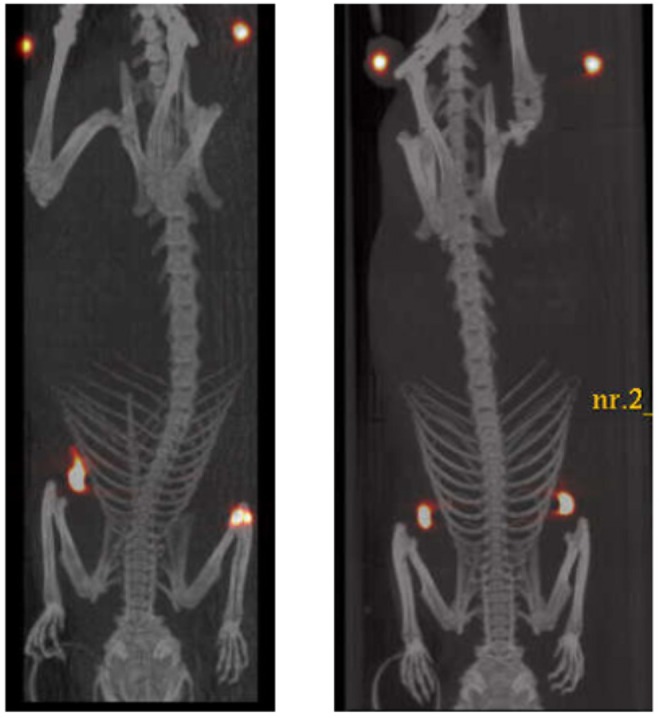
µPet/CT superimposed images registered 40 h after the injection of ^64^Cu-HAC in the mammary glands of two adult female rats. In the upper part of the images, near hind legs, two objects with high density and imbibed with ^64^Cu are located, as reference points for accurate overlapping of PET and CT images.

The test demonstrated that the tracer remained located in the mammary glands for at least 40 h (Fig. 4).

**Figure 5:**
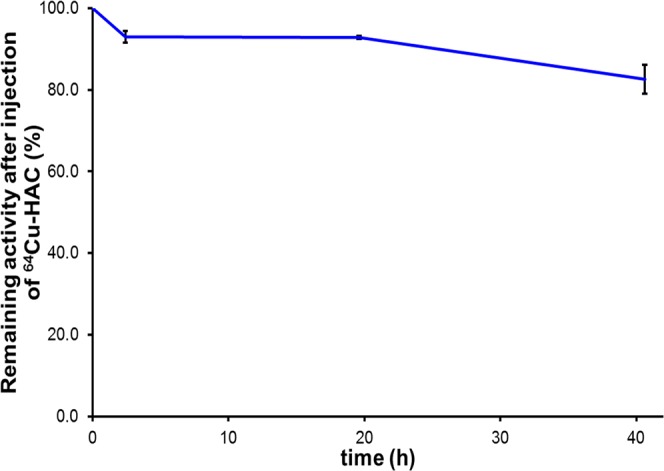
Relative uptake value (mean ± SD) referred to the ^64^Cu-HAC activity of the dose injected in the mammary glands of two adult female rats.

After 2.5 h from the injection, the activity in the glands was 94 ± 1% of the initial one and remained constant for the next 20 h. Forty hours after the injection the signal persisted, even if slightly reduced (85%) (Fig. 5).

The initial radioactivity release, traceable in the kidneys and in the bladder, can be explained with the well-known loose interaction between Cu and albumin, which is at the base of the physiological role played by this plasmatic protein as a transporter of this metal^28^. In fact, concurrently to the ^64^Cu-HAC labeling by DOTA chelation, a little amount, about 6%, of ^64^Cu binds aspecifically to albumin, and is released *in vivo* during the first 2.5 h after the administration. The residual unconjugated albumin, approx. 10–20% by weight of HAC, as shown by the MALDI-TOF study, can account for this aspecific binding.

On the other hand, the slight and poorly reproducible decline of radioactivity in the glands after 40 h, i.e. after more than three ^64^Cu half-lives, is possibly due to the approximation of the detection limits of the µPET apparatus used.

Thus, the evidences gathered until now justify further studies for the development of this new investigational medicinal product with the final objective of testing this imaging agent in an authorized clinical trial to study its application in the ROLL procedure.

Besides, seen the persistence of gamma signal for several days, due to the use of ^177^Lu and ^111^In, the application of HAC has also been hypothesized in other fields of surgical oncology, as for example the localization of lateral cervical lymph nodes in patients with head-neck tumors.

## Conclusions

The new insoluble conjugate of HA and *p*-SCN-Bn-DOTA, here described, can be produced by a one-pot reaction in aqueous medium that guarantees high purity and good reaction yield. The product radiolabeled with ^177^Lu or ^111^In shows radiochemical purity >90% and no sign of radiolysis or biodegradation after storage in plasma for 6 days at 37 °C. In an *in vivo* µPET/CT study on adult female rats, HAC labeled with ^64^Cu remained located in the mammary glands without spread in healthy tissues or drainage in the lymphatic circulation, for at least 40 h, a time consistent with the not long half-life of the positron-emitter used. Therefore, the evidences on this derivative, gathered by *in vitro* and *in vivo* preliminary studies, support its potential use in human ROLL procedure for the detection of non-palpable mammalian cancerous lesions.

HAC allows to overcome the main drawback of ROLL using ^99m^Tc, i.e. the scheduling of patient for localization procedure and subsequent surgery, due to the short half-life of this radionuclide.

By replacing ^99m^Tc with longer lived radionuclides, the previously published evaluations about safety^10^ do not change, since the gamma emissions of either ^177^Lu or ^111^In are similar to that of ^99m^Tc. In addition, the same radioactivity, or maybe less (due to the longer half-life), might be injected in association with the described microdispersed albumin derivative.

Moreover, the existing gamma probes available in breast surgery are able to detect signal from ^177^Lu and ^111^In. Regarding ^177^Lu, its 160 keV gamma emission makes it similar to ^99m^Tc, the detection efficiency for both these radionuclides being set >60% by the manufacturer.

The proposed use of ^177^Lu to label the albumin derivative is due to its favourable physical characteristics. The 6.64 days half-life, the low β^−^ energy (β^−^ max = 0.5 MeV) accompanied by imageable gamma emission may allow to inject the radiopharmaceutical into the occult lesion few days before surgery. In this scenario the short penetration range (Rmax = 2 mm) of the beta particles might also be useful to destroy tumor cells surrounding the lesion, entailing no harm to the patient.

If further studies confirm the preliminary proofs, this product will make the ROLL procedure more flexible, allowing to schedule surgical interventions on several days. Moreover, the localization procedure and its verification performed as an outpatient exam in the days preceding surgery will reduce the hospitalization time and cost, increasing the number of women who will benefit from it, with a substantial social impact.

Although this new imaging agent has been prepared to improve the efficacy, safety and health-care system results of the image-based surgical intervention in breast cancer, it might also be suitable for other radio-guided surgical procedures in different fields of oncology, as for example in solitary pulmonary nodules.

## Materials and Methods

### Materials

See Supplementary Information.

### Human albumin purification

0.5 mL of 200 mg/mL human albumin (HA) aqueous solution and 3 mL of 0.9% NaCl aqueous solution were placed in an Amicon Ultra-4 ultrafiltration system (cut-off 10,000 NMWL) and the device was gently stirred by oscillation for 5 min, then the mixture was ultrafiltered at 7400 *g* for 15 min. The washing step was repeated at least 3 times, or until the ultrafiltrate no longer showed the spectrophotometric absorbance at 280 nm due to *N-*acetyl tryptophan.

To the purified human albumin (HAP) solution, 0.9% NaCl was added to obtain a 2 mL HAP stock solution containing approx. 50 mg/mL of HA. The concentration of HAP stock solution was carefully determined by UV absorbance at 280 nm versus a pure HA standard solution. The HAP stock solution was stored in the fridge.

### HAC preparation

5.00 mg of *p*-SCN-Bn-DOTA, 0.5 mL of HAP stock solution (molar ratio 20:1), 60 μL of 0.1 M carbonate buffer pH 9.0 and 0.5 mL of 0.9% NaCl were mixed in a 1.5 mL polypropylene tube, which was heated at 40 °C, under 300 rpm continuous orbital shaking, for 16 h in a thermoshaker (SC25XT, Torrey-Pines Scientific, US). The reaction product was purified by three washings with saline and two washings with 1 M sodium acetate buffer pH 5.0, each one followed by ultrafiltration on a 10,000 NMWL ultrafiltration device at 7400 *g* for 15 min, until complete disappearance of the UV absorbance of the macrocyclic reagent in the ultrafiltrate. The concentrated product, approx. 0.5 mL of suspension containing HAC in 1 M sodium acetate buffer pH 5.0, was diluted to 1.5 mL and stored in the fridge; alternately, the suspension was washed three times with UHQ water by ultrafiltration, and freeze-dried.

### IR analysis and Differential Scanning Calorimetry (DSC)

See Supplementary Information.

### MALDI-TOF analysis

1 mg of HAC was dissolved in 1 mL of H_2_O containing 0.1% of trifluoroacetic acid (TFA) and further diluted 1:10 (v/v) with 0.1% TFA. The sample solutions were deposited by a sinapinic acid matrix, using the traditional MALDI “Dried Droplet” deposition technique. For further details see Supplementary information.

### NanoLC-MS/MS sample preparation and data processing

HAP or HAC samples were digested with trypsin and the resulting peptides were analyzed by reversed-phase chromatography using Ultimate 3000 RSLC HPLC system connected online to an Orbitrap Fusion Tribrid (Thermo Fisher Scientific, US) operating in positive ionization mode applying a hybrid method: high-low-high resolution DDA. For further details see Supplementary information.

### Spectrophotometric determination of HAC degree of substitution (DS)

A stock solution containing 67.62 μmol/L of Pb(II) and 140 μmol/L of arsenazo III (AA) in 0.15 M AcONH_4_ buffer pH 7.0 was prepared. Such stock solution, Pb(II)-AA, must be stored in the dark, at 2–6 °C, and used at room temperature within 24 h since it was prepared. A 0.344 mM stock solution of DOTA in 0.15 M AcONH_4_ buffer pH 7.0 was prepared. For each calibration curve, six standard solutions were prepared, by mixing 3.4 mL of Pb(II)-AA stock solution, 200 μL of 1 M NaCl in ammonium acetate and a variable volume of DOTA stock solution (0–500 μL, corresponding to DOTA concentrations from 0 to 0.041 mM), and finally adding 0.15 M AcONH_4_ buffer pH 7.0 to a final volume of 4.2 mL. The absorbance at 656 nm at room temperature was determined on each standard solution, protected from light, 10 min after its preparation. The reading was corrected by subtracting the absorbance of a solution constituted of 4.0 mL of 0.15 M AcONH_4_ buffer pH 7.0 and 200 μL of 1 M NaCl. For the sample solution, 0.90 to 1.10 mg of HAC were dispersed in a mixture composed of 3.4 mL of Pb(II)-AA stock solution, 200 μL of 1 M NaCl, and 600 μL of 0.15 M AcONH_4_ buffer pH 7.0, and its absorbance at 656 nm at room temperature was determined, protected from light, 10 min after its preparation. Results are expressed as μmoles of DOTA/mg HAC or as DS = μmoles DOTA/μmoles HA. DS is calculated through the following Eq. (1):

1 $$DS=\frac{M{W}_{HA}}{\frac{\mu gHAC}{\mu molesDOTA}-M{W}_{DOTANCS}}$$

where: *MW*_*HA*_ = HA molecular weight; *µgHAC* = μgrams of HAC freeze-dried sample; *µmolesDOTA* = μmoles of HA-conjugated DOTA calculated from the calibration curve; *MW*_*DOTANCS*_ = *p*-SCN-Bn-DOTA molecular weight.

### HAC particle size distribution and shape

See Supplementary Information.

### Complexation of HAC with ^175^Lu and ICP-OES determination

The amount of ^175^Lu was determined by ICP-OES (iCAP 7000 series, Thermo Scientific, US) at 261.542 nm through a 6-point calibration curve. For further details, see Supplementary information.

### HAC radiolabeling

Radiolabeling was carried out with both ^111^In and ^177^Lu at the specific activity of 1 mCi/mg, using HAC (10–20 mg/mL) suspended in 1 M sodium acetate pH 5.0. A suitable volume of ^177^LuCl_3_ or ^111^InCl_3_ in 0.05 N HCl, corresponding to the calculated activity, was added in a sterile polypropylene tube already containing the HAC suspension in sodium acetate, which, after mixing, was heated to 90 °C for 30 min.

### Radiochemical purity (RCP)

See Supplementary Information.

### Procedure for the evaluation of microbial contamination

See Supplementary information.

### Stability of HAC labeled with ^177^Lu and ^111^In in saline at 37 °C

120 μL of HAC suspension (containing 2 mg of HAC) in 1 M sodium acetate pH 5.0 were labeled with ^177^Lu or ^111^In in saline to obtain, after dilution with saline to 240 μL, a 2 mCi final suspension. The stability at 37 °C of radiolabeled HAC in saline was evaluated by measuring its RCP 6, 24, 48, and 144 h after labeling.

### Stability of HAC labeled with ^177^Lu or ^111^In in human plasma at 37 °C

1 mL of 1:1 v/v human plasma/ultrapure water mixture was added to 1 mL of 16.7 mg/mL radiolabeled HAC suspension (see HAC radiolabeling paragraph) in 1 M sodium acetate pH 5.0, followed by incubation at 37 °C in a thermostatic bath. The RCP was assessed after 24, 48, and 144 h.

### µPET and µCT imaging

HAC was labeled with ^64^Cu, a positron-emitting radioisotope (t_1/2_ = 12.7 h and decay properties β^−^ 38.4%, β^+^ 17.8%) to evaluate its retention in the mammary gland tissue through positron emission tomography (PET) studies. ^64^CuCl_2_ was prepared according to the method described in Matarrese *et al*.^27^. The solid target irradiation was performed using a PETtrace cyclotron (GE Healthcare, US), while production, dissolution, purification and recovery of ^64^Cu were performed into the automated module ALCEOHALOGEN (Comecer S.p.A., IT). The ^64^Cu obtained was used to label HAC according to the radiolabeling procedure described in the previous section. The radiochemical purity of labeled HAC was assayed by iTLC. The radiolabeling yield was 100% and the specific activity of the final product was 0.9 mCi/mg. *In vivo* tests were performed at the Radiochemistry Department of NSA Nuclear Specialists Associated Srl (Caronti, Ardea, IT) and the animals were used for scientific scope according to the Italian Health Ministry authorization DM 112/2009-A. The experiments on animals were performed in accordance with Italian regulations and EU Directive for the protection of animals used for scientific purposes.

Two female adult rats were housed in a controlled environment with a 12-h light/dark cycle and maintained on Mucedola Certified Rodent Diet (4RF21 GLP Certificate) and water *ad libitum*. The animals were anesthetized with an oxygen/isoflurane gas mixture and injected in the thoracic left and right mammary glands with a ^64^Cu-HAC suspension in sodium acetate pH 5.0. ECG and respiration were constantly monitored during the analysis. A whole body μPET imaging scan was performed for 30 min 0, 2.5, 20, 40 h after the radiotracer injection. PET imaging was performed using a µPET scanner (Explore Vista, GE Healthcare, US) and the PET scans were reconstructed using the ordered subset expectation maximization (OSEM) algorithm. A μCT (Computed Tomography) imaging scan was also carried out using a µCT scanner (Explore Locus, GE Healthcare, US) and the μCT projection images were acquired over a rotation of about 200° at 80 kVp, 450 mA, 90 μm resolution. The μCT images were reconstructed to give a volume data-set. After the data-set reconstruction, PET and CT images were overlapped to quantify ^64^Cu-HAC in the mammary glands, by drawing regions of interest (ROIs) of the target sites.

### Statistics

Data were analyzed using Systat 13. Statistical significance was established at *p* < 0.05.

## Supplementary information

Supplementary Information
