# Author Correction: A new microdispersed albumin derivative potentially useful for radio-guided surgery of occult breast cancer lesions

**DOI:** 10.1038/s41598-020-60471-y

**Published:** 2020-03-10

**Authors:** Gabriele Caviglioli, Marco Chinol, Sara Baldassari, Lucia Garaboldi, Guendalina Zuccari, Andrea Petretto, Giuliana Drava, Chiara Sinico, Giovanni Paganelli

**Affiliations:** 1 Department of Pharmacy (DIFAR), University of Genova, 16148 Genova, Italy; 2 Division of Nuclear Medicine, European Institute of Oncology, 20141 Milano, Italy; 3 Core Facilities-Proteomics Laboratory, Istituto Giannina Gaslini, 16147 Genova, Italy; 4 Department of Life and Environmental Sciences, University of Cagliari, 09124 Cagliari, Italy; 5 Istituto Scientifico Romagnolo per lo Studio e la Cura dei Tumori, IRST-IRCCS, Meldola. via P. Maroncelli 40, Meldola, Italy

Correction to: *Scientific Reports* 10.1038/s41598-019-42014-2, published online 04 April 2019

In the original version of this Article, Giovanni Paganelli was incorrectly affiliated with ‘Division of Nuclear Medicine, European Institute of Oncology, 20141, Milano, Italy’. The correct affiliation is listed below.

Istituto Scientifico Romagnolo per lo Studio e la Cura dei Tumori, IRST-IRCCS, Meldola. via P. Maroncelli 40, Meldola, Italy

This error has now been corrected in the PDF and HTML versions of the Article and in the accompanying Supplementary Information file.

