# Supplementary material for: A new microdispersed albumin derivative potentially useful for radio-guided surgery of occult breast cancer lesions
Source: Sci Rep. 2019 Apr 4;9:5623. doi: 10.1038/s41598-019-42014-2 (PMC6449347; doi:10.1038/s41598-019-42014-2)
Supplement: Supplementary file 1 — Supplementary Information [file 41598_2019_42014_MOESM1_ESM.pdf]

A new microdispersed albumin derivative potentially useful for radio-guided surgery of occult breast cancer lesions

Gabriele Caviglioli[1]*, Marco Chinol[2]*, Sara Baldassari[1], Lucia Garaboldi[2], Guendalina Zuccari[1], Andrea Petretto[3], Giuliana Drava[1], Chiara Sinico[4], Giovanni Paganelli[2]

SUPPLEMENTARY INFORMATION

MATERIALS AND METHODS

Materials

Human albumin standard (fatty acid free, globulin free), lead standard for AAS TraceCERT®, arsenazo III, $LuCl_3$ and lutetium ICP/DCP standard solution (Sigma-Aldrich, D); 50 mL 20% Albutein® (Alpha Therapeutic S.p.A, IT); DOTA and *p*-SCN-Bn-DOTA (Macrocyclics, US); sodium chloride 0.9% saline (Eurospital, IT); 65% $^{177}LuCl_3$ (Perkin Elmer, US); $^{111}InCl_3$ (Covidien, US); trypsin (Promega Corporation, US); Amicon® Ultra-4 ultrafiltration devices, cut-off 10,000 NMWL (Millipore Merck, US). Only analytical grade reagents were used.

IR analysis

IR spectra were recorded by a Perkin Elmer 2000 FT-IR spectrometer (US) from lyophilized HAP and HAC samples in KBr tablets.

Differential Scanning Calorimetry (DSC)

Accurately weighed samples in the range of 2.5 ± 0.5 mg were subjected to a thermal analysis from 30 to 250 °C at a heating rate of 10 °C/min, under nitrogen atmosphere in an open aluminum pan, by a Perkin–Elmer DSC-7 (US).

MALDI-TOF analysis

1 mg of HAC was dissolved in 1 mL of H_2O containing 0.1% of trifluoroacetic acid (TFA) and further diluted 1:10 (v/v) with 0.1% TFA. The sample solutions were deposited by a sinapinic acid matrix, using the traditional MALDI "Dried Droplet" deposition technique. For the matrix preparation, 10 mg of each matrix were dissolved in 1 mL of H_2O/ACN solution (50:50 v/v) containing 0.1% TFA. The samples deposited on the MALDI plate were analyzed using a MALDI TOF-TOF instrument (UltrafleXtreme, Bruker Daltonics, US) operating in linear mode with positive ion detection. The instrumental conditions optimized for the analysis were: IS1 = 25 kV; IS2 = 23.3 kV; Lens = 6.5 kV; PIE = 450 ns; laser power = 60%.

NanoLC-MS/MS sample preparation and data processing

HAP or HAC samples were treated using a solution of 2% (w/v) SDC, 10 mM TCEP, 40 mM CAA, 100 mM Tris pH 8.5. Afterwards, the samples were diluted 1:1 and trypsin was added for a 3-h digestion at 37 °C. Then, the samples were purified on SDB-RPS Stage Tips with a solution of isopropanol and 1% TFA. The peptides were separated and analyzed by reversed-phase chromatography (EASY-Spray column PepMap C18 2 µm, 50 cm x 75 µm, Thermo Fisher Scientific, US), by increasing the organic solvent at a flow rate of 250 nL/min

with a nonlinear gradient of 2-80 % solution B (80% acetonitrile and 20% H_2O, 5% DMSO, 0.1% TFA) in 70 min using Ultimate 3000 RSLC HPLC system connected online to an Orbitrap Fusion Tribrid (Thermo Fisher Scientific, US) operating in positive ionization mode applying a hybrid method: high-low-high resolution DDA. Briefly, MS scans were acquired in the Orbitrap with a resolution of 120,000. Precursors of z = 2-5 were selected for 1 second of cycle time, dynamic exclusion was set to 25 seconds. Charge states 3−5 with precursor intensity > 500,000 were scheduled for analysis by a fast HCD/FT. The charge-state 3−5 ions with intensity < 500,000 and the doubly charged ions were scheduled for analysis by CID/IT. Moreover, a second method was applied, using HCD/FT scan with a collision energy of 28%, maximum injection time of 55 ms and 15,000 of resolution. Raw MS files were processed with MaxQuant software using Human Albumin database. Cysteine carbamidomethylation as fixed modification and acetylation of protein *N*-terminal, methionine oxidation, deamidation (N, Q), and *"PM_residuo"* as variable modifications were used.

HAC particle size distribution and shape

Particle size distribution was assessed by analyzing samples (16.5 mg/mL) from 5 microbatches of HAC, using a laser diffraction analyzer (Mastersizer, Malvern, UK). The samples were analyzed as such and after sonication at 20% intensity for 5 minutes, using Mie procedure as scattering model and normal sensitivity analysis. Some samples were analyzed after three cycles of heating to 90 °C and rapid cooling to 5 °C, others after injection through a 21G needle. Particle size and morphology were assessed on a HAC aqueous dispersion (16.5 mg/mL) using an automated imaging system (Morphologi, Malvern, UK).

Complexation of HAC with ^{175}Lu and ICP-OES determination

8.0 mg of HAC, accurately weighed, were dispersed in 400 µL of 1 M sodium acetate pH 5.0 and a solution containing 4.0 mg of $LuCl_3$ dissolved in HCl 0.05 N was added to the dispersion. The complexation was carried out in a thermoshaker at 90 °C for 30 min under 400 rpm orbital stirring. The cooled suspension was washed first with 0.9% NaCl, then with deionized water to remove the excess of $LuCl_3$. The ICP-OES (iCAP 7000 series, Thermo Scientific, US) determinations of ^{175}Lu were performed at the wavelength of 261.542 nm through a 6-point calibration curve obtained with aqueous standard solutions containing up to 3 µg/mL of ^{175}Lu standard for ICP. The amount of ^{175}Lu was determined in an accurately weighed HAC-^{175}Lu sample, in the range 0.90 to 1.10 mg, dissolved in 1 mL of HNO_3 65% and kept at room temperature for 1 h; before the analysis, the sample was diluted with 1 mL of UHQ water.

Radiochemical purity (RCP)

Each radiolabeled sample was analyzed in triplicate for radiochemical purity by instant thin-layer chromatography (iTLC). 0.1 mL of 16.7 mg/mL radiolabeled suspension of HAC with ^{111}In or ^{177}Lu in sodium acetate pH 5.0 (specific activity 1 mCi/mg) was mixed with 0.1 mL of a 2.5 mM solution of diethylene-triamine-pentaacetic acid (DTPA) pH 5. 0.5 µL of the radioactive suspension were deposited on an iTLC-SG support and developed by ascending chromatography, with 0.9% NaCl as the mobile phase. Under these conditions, radiolabeled HAC does not migrate, while the free radionuclide, potentially present, complexed by DTPA moves to the solvent front. The radiochromatographic profile was measured by an autoradiographic equipment that uses a high performance storage phosphor screen (Packard BioScience Cyclone, US), and the radiochemical purity was consequently calculated.

Procedure for the evaluation of microbial contamination

For microbial contamination evaluation, performed according to fill-test guidelines in an internal microbiology unit, 1.5 mL of HAC suspension in 1 M sodium acetate pH 5.0 were incubated in a thermostated oven (series 6000, Heraeus Instruments, D) at 37 ± 1 °C for 14 days. In case the sample became turbid, 1 mL of the suspension was plated in a Petri dish to quantitatively evaluate the microbiological growth. The HAC batch used for the *in vivo* test was submitted to this test primarily.

SUPPLEMENTARY TABLES AND FIGURES

Table S1. Radiochemical purity of radiolabeled (1 mCi/mg) HAC batches.

**Batch #**	**mg HAC/mL**	**Radionuclide**	**RCP (%)**
A	16.7	^{111}In	95.0
B	16.5	^{111}In	98.3
A	16.4	^{177}Lu	96.6
B	16.6	^{177}Lu	99.6

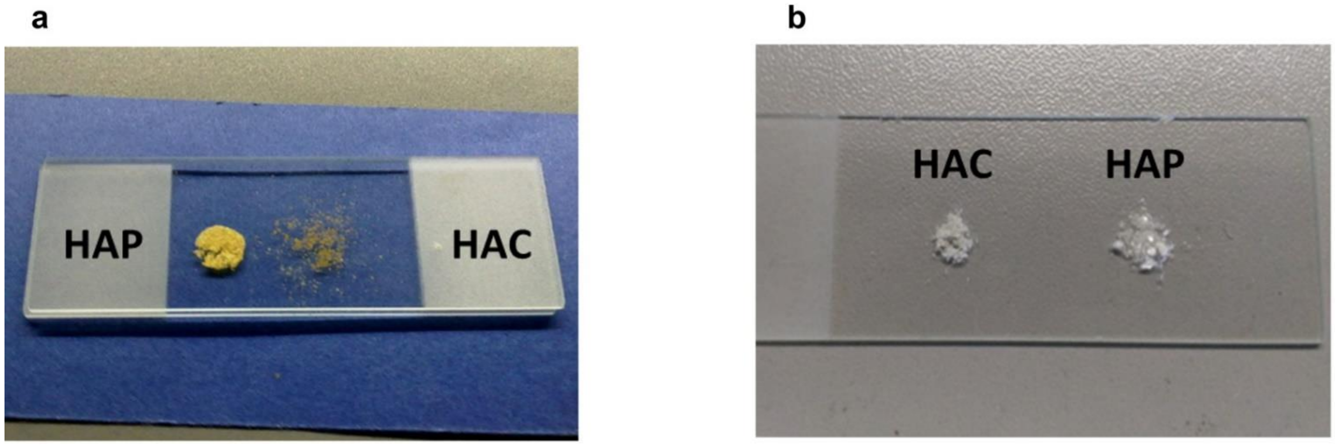

Figure S1. *Comparison of HAC and HAP morphological changes after (**a**) DSC and (**b**) lyophilization.*

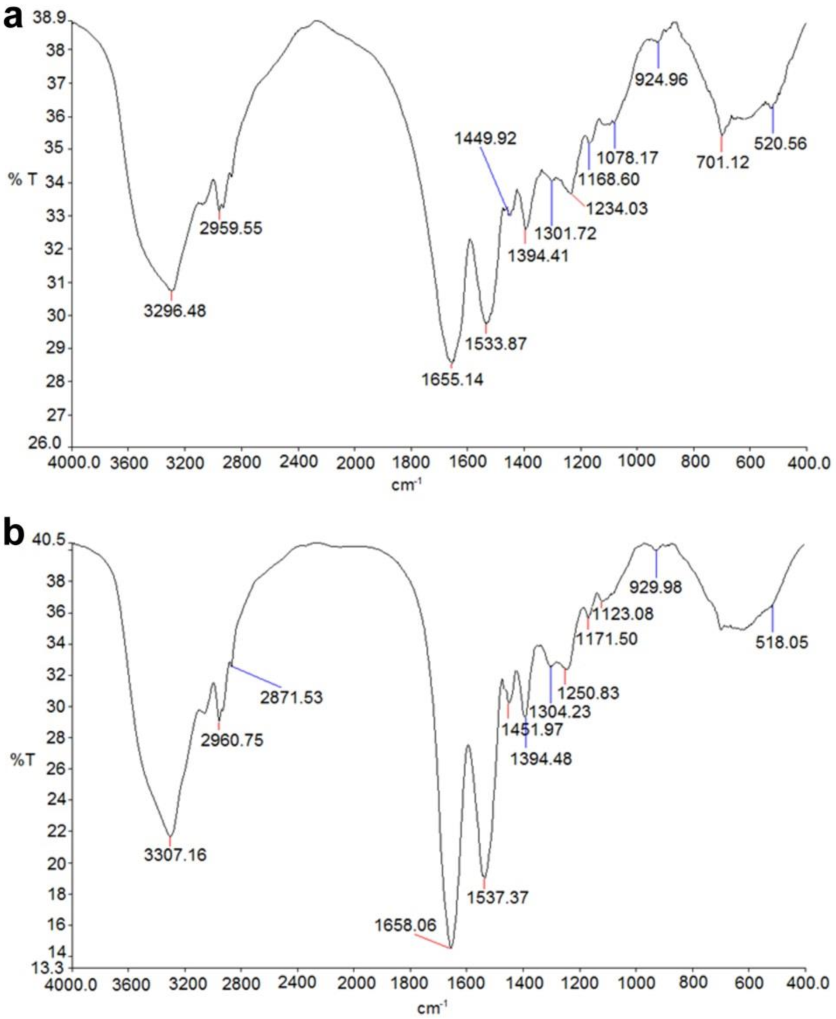

Figure S2. *Comparison between IR spectra of (**A**) freeze-dried HAC and (**B**) freeze-dried HAP in KBr.*

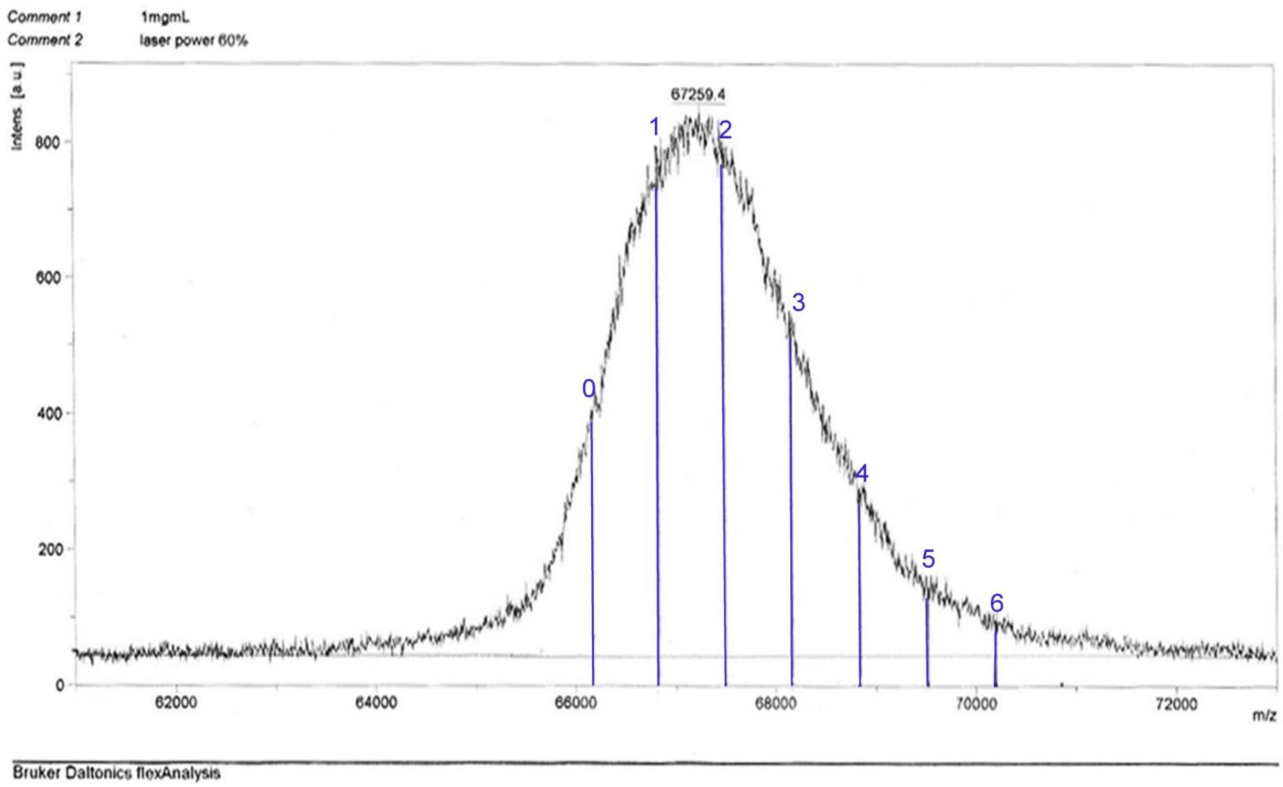

Figure S3. *MALDI-TOF spectrum of HAC Batch A with overwritten m/z values of HAC containing from 0 to 6 p-SCN-Bn-DOTA residues.*

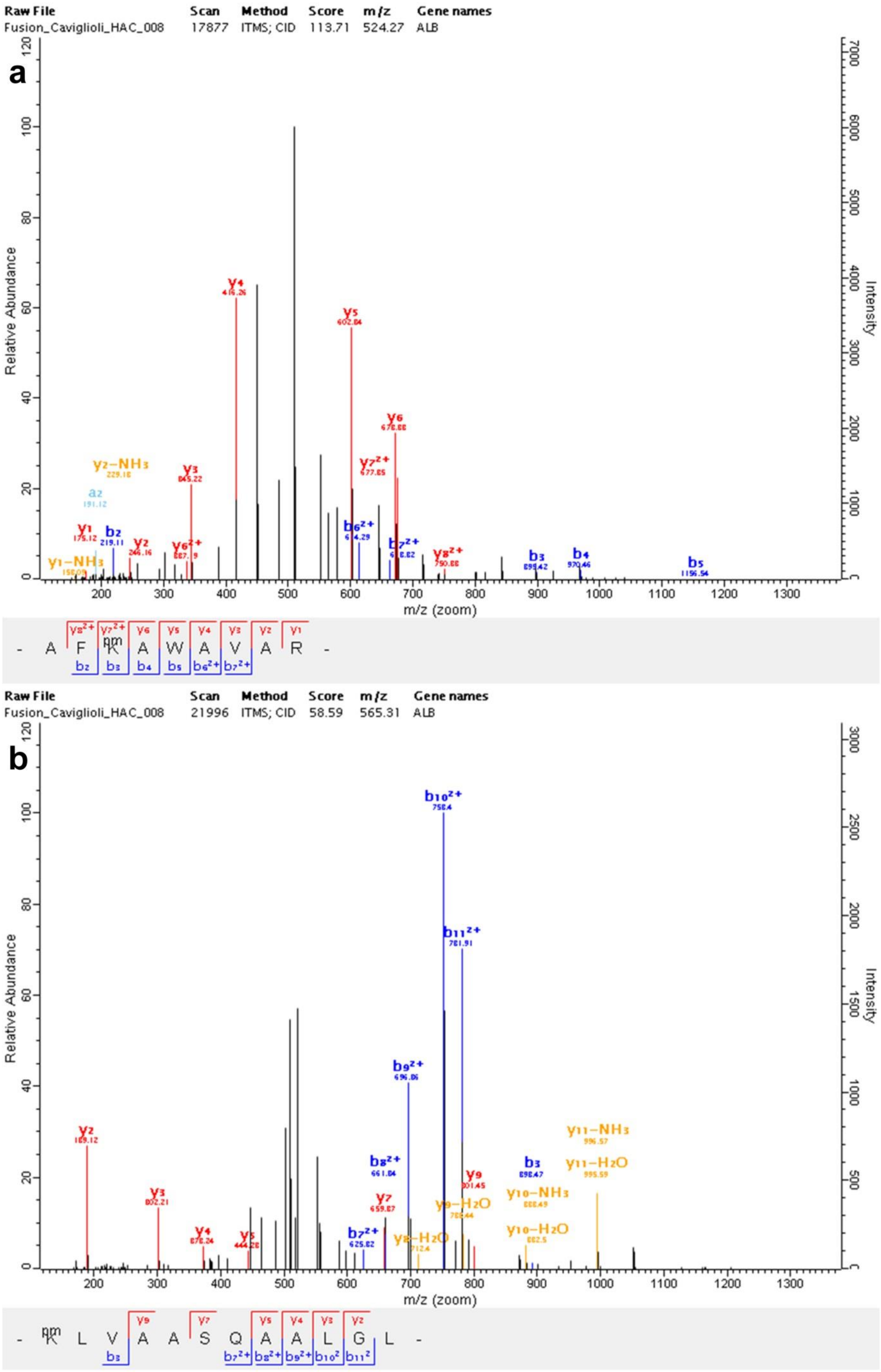

Raw File Scan Method Score m/z Gene names
Fusion_Caviglioli_HAC_008 17877 ITMS; CID 113.71 524.27 ALB
a
Relative Abundance
Intensity
m/z (zoom)
- A F K A W A V A R -
Raw File Scan Method Score m/z Gene names
Fusion_Caviglioli_HAC_008 21996 ITMS; CID 58.59 565.31 ALB
b
Relative Abundance
Intensity
m/z (zoom)
- K L V A A S Q A A L G L -

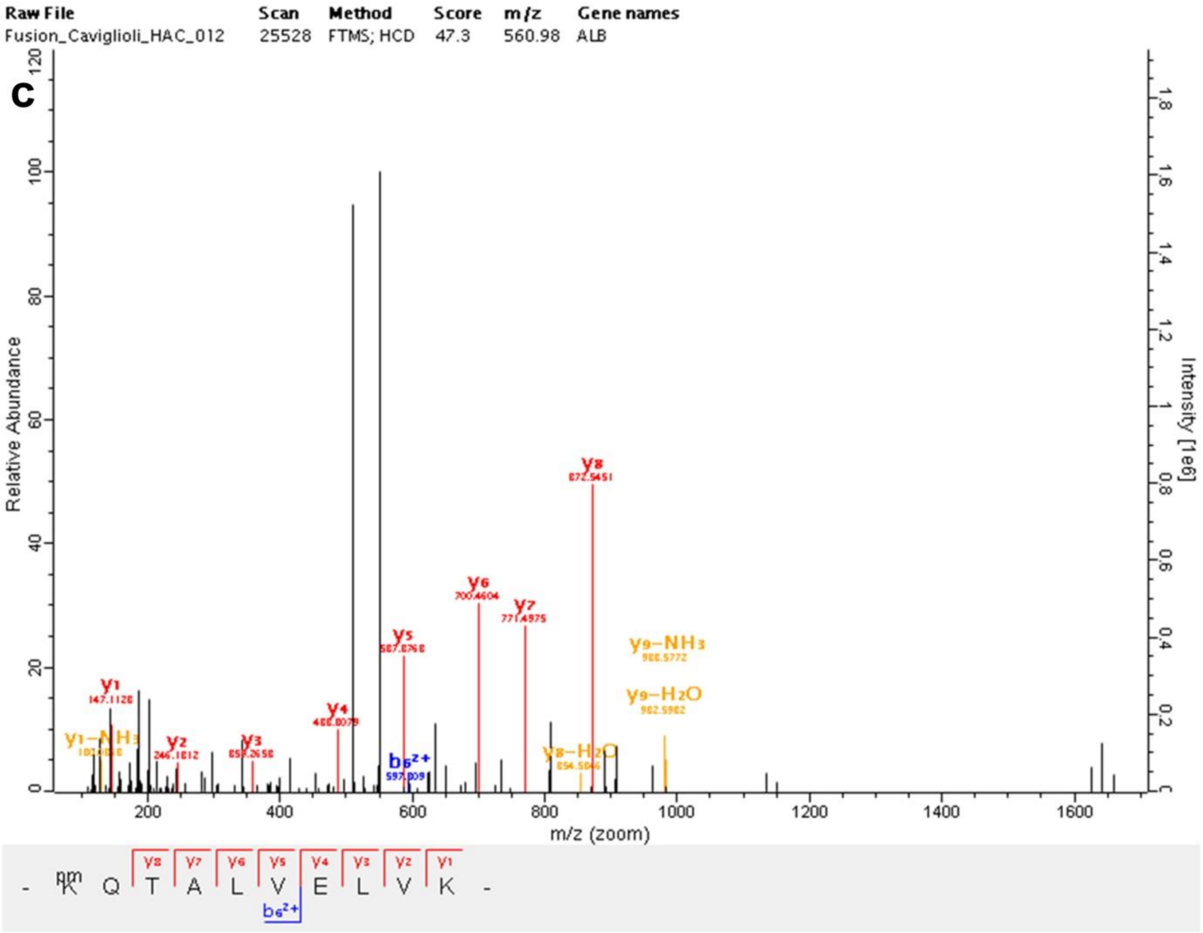

Raw File Scan Method Score m/z Gene names
Fusion_Caviglioli_HAC_012 25528 FTMS; HCD 47.3 560.98 ALB
c
Relative Abundance
Intensity [1e6]
m/z (zoom)
y8
y6
y7
y5
y9-NH3
y9-H2O
y1
y4
y2
y3
b6²+
y8-H2O
- K Q T A L V E L V K -

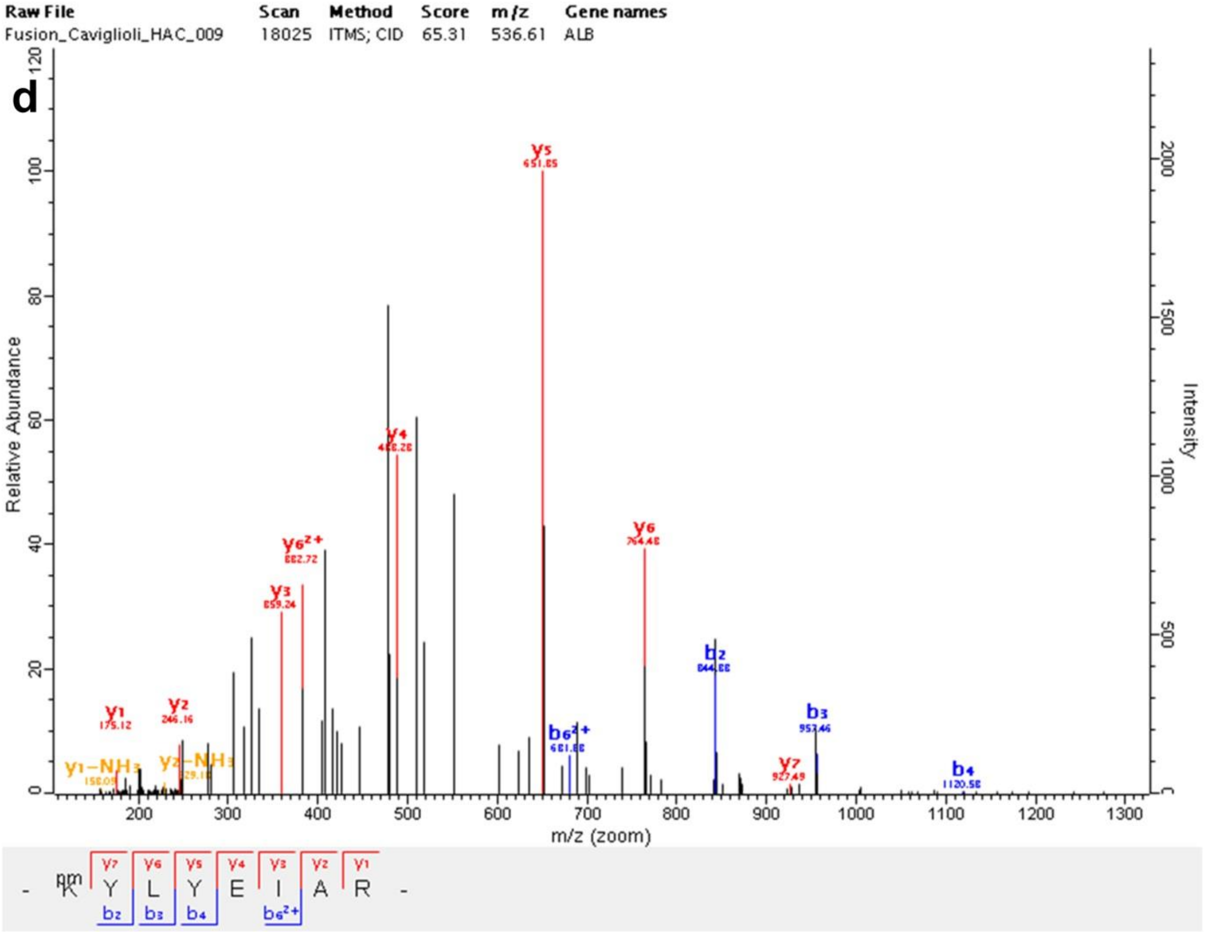

Raw File Scan Method Score m/z Gene names
Fusion_Caviglioli_HAC_009 18025 ITMS; CID 65.31 536.61 ALB
d
Relative Abundance
Intensity
m/z (zoom)
y5
y4
y6²+
y3
y6
b2
b3
b6²+
y1
y2
y7
b4
- K Y L Y E I A R -

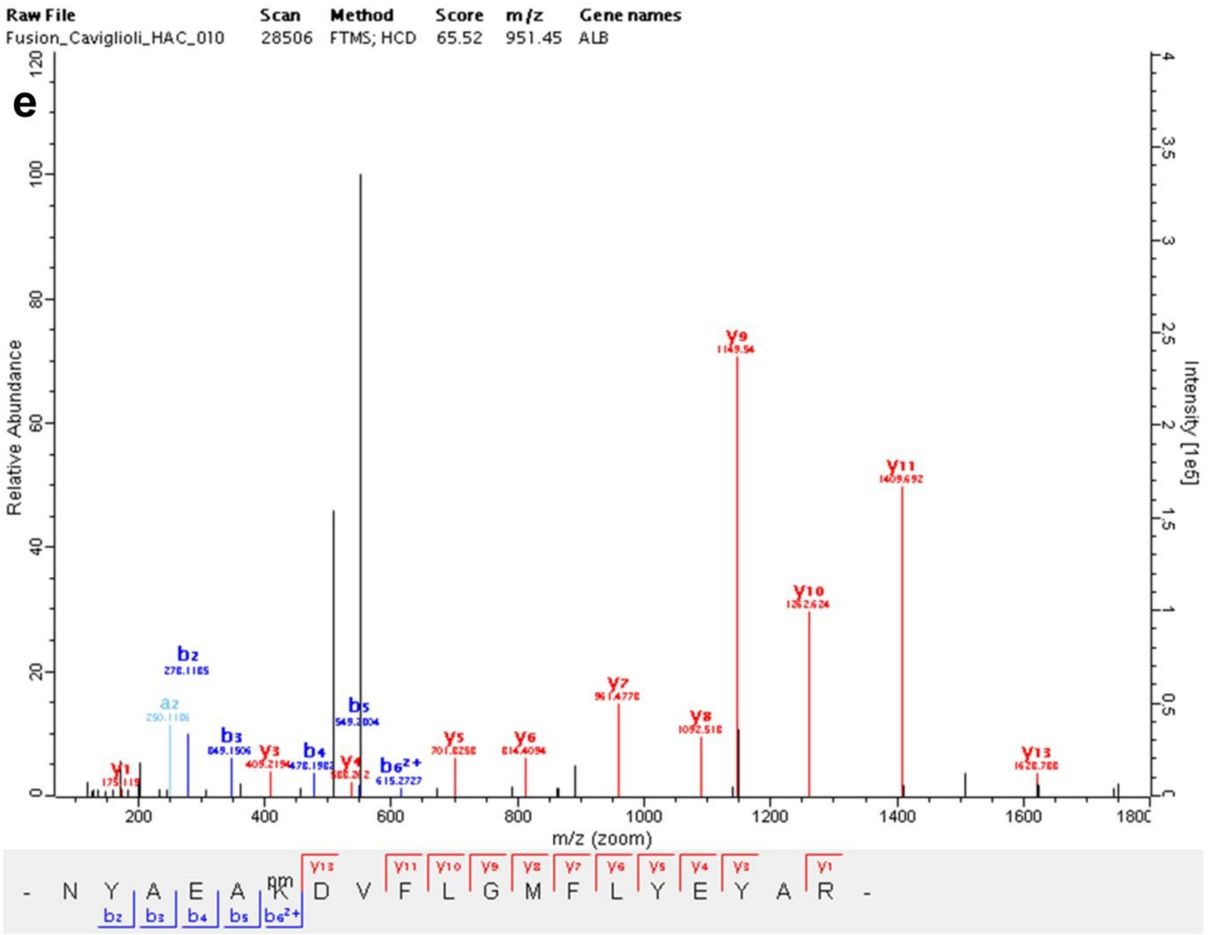

Figure S4. *Annotated fragment spectra of five peptides identified for the presence of the p-SCN-Bn-DOTA residue. The corresponding sequence is displayed under each MS/MS spectrum and shows the position of the adduct (pm) on the lysine site (K). (**a**) AFKAWAVAR; (**b**) KLVAASQAALGL; (**c**) KQTALVELVK; (**d**) KYLYEIAR; (**e**) NYAEAKDVFLGMFLYEYAR.*

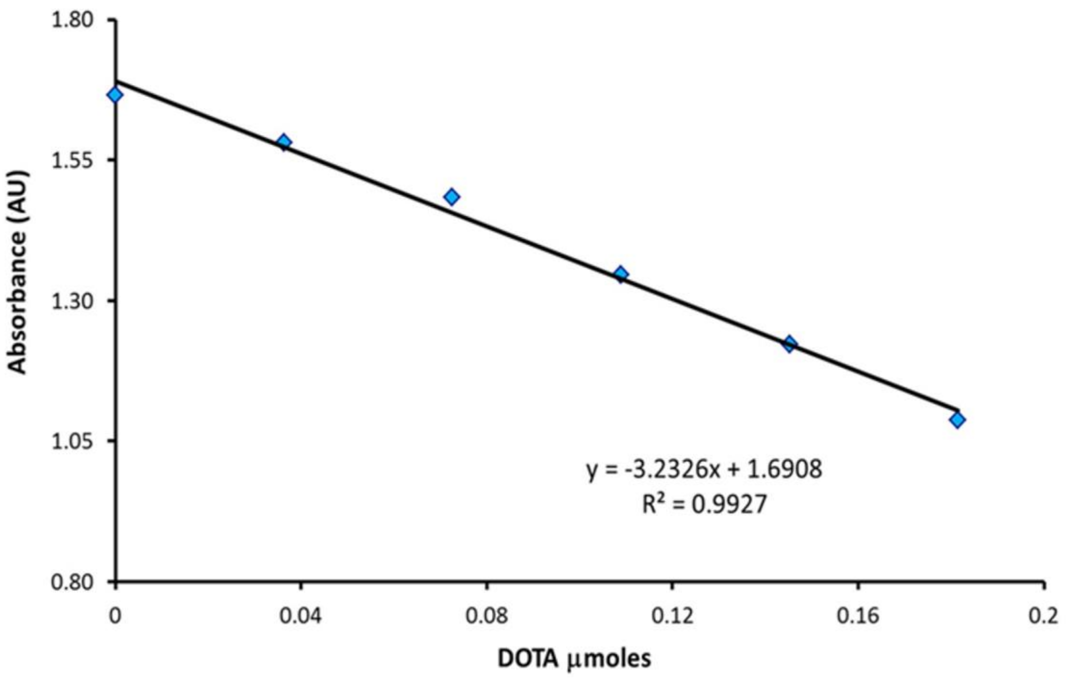

Figure S5. *Regression line for spectrophotometric determination of HAC degree of substitution.*

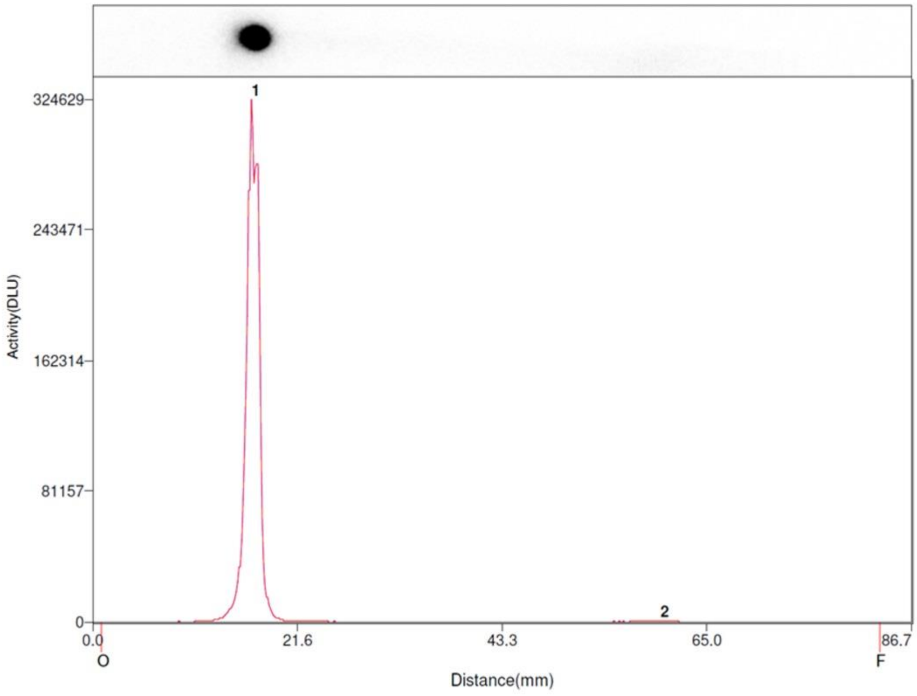

Lane #2
Background Subtraction: Regions = 436,544 DLU /mm2
Lane Label: C8

ID	RF	Gross DLU	Background Subtract	Net DLU	Net % Sum
1 - Prof	0,2	3.080.590,5	89.692,1	2.990.898,4	99,6
2 - Prof	0,7	85.307,0	72.505,6	12.801,4	0,4
1-b - Prof		739,2			
Lane		3.304.712,2	378.640,4	2.926.071,8	
UnRes		138.814,6	216.442,6	-77.628,0	

Figure S6. *Radio-iTLC chromatogram of HAC (16.4 mg/mL) labeled with 1 mCi/mg of ^{177}Lu (RCP 99.6%).*
